# Myrislignan targets extracellular signal-regulated kinase (ERK) and modulates mitochondrial function to dampen osteoclastogenesis and ovariectomy-induced osteoporosis

**DOI:** 10.1186/s12967-023-04706-2

**Published:** 2023-11-22

**Authors:** Tao Yang, Weiwei Chen, Kai Gan, Chaofeng Wang, Xiaoxiao Xie, Yuangang Su, Haoyu Lian, Jiake Xu, Jinmin Zhao, Qian Liu

**Affiliations:** 1 Guangxi Key Laboratory of Regenerative Medicine, Orthopaedic Department, The First Affiliated Hospital of Guangxi Medical University, Nanning, 530021 Guangxi China; 2 Collaborative Innovation Centre of Regenerative Medicine and Medical BioResource Development and Application Co-constructed by the Province and Ministry, Life Sciences Institute, Guangxi Medical University, Nanning, 530021 Guangxi China; 3 School of Biomedical Sciences, the University of Western Australia, Perth, WA 6009 Australia

**Keywords:** ERK, Mitochondria, Myrislignan, LM22B-10, Osteoclast, Osteoporosis

## Abstract

**Background:**

Activated osteoclasts cause excessive bone resorption, and disrupt bone homeostasis, leading to osteoporosis. The extracellular signal-regulated kinase (ERK) signaling is the classical pathway related to osteoclast differentiation, and mitochondrial reactive oxygen species are closely associated with the differentiation of osteoclasts. Myrislignan (MRL), a natural product derived from nutmeg, has multiple pharmacological activities; however, its therapeutic effect on osteoporosis is unclear. Here, we investigated whether MRL could inhibit osteoclastogenesis and bone mass loss in an ovariectomy mouse model by suppressing mitochondrial function and ERK signaling.

**Methods:**

Tartrate-resistant and phosphatase (TRAP) and bone resorption assays were performed to observe the effect of MRL on osteoclastogenesis of bone marrow macrophages. MitoSOX RED and tetramethyl rhodamine methyl ester (TMRM) staining was performed to evaluate the inhibitory effect of MRL on mitochondria. Quantitative reverse transcription-polymerase chain reaction (qRT-PCR) assay was performed to detect whether MRL suppressed the expression of osteoclast-specific genes. The impact of MRL on the protein involved in the mitogen-activated protein kinase (MAPK) and nuclear factor-κB pathways was evaluated using western blotting. In addition, a specific ERK agonist LM22B-10, was used to revalidate the inhibitory effect of MRL on ERK. Finally, we established an ovariectomy mouse model to assess the therapeutic effect of MRL on osteoporosis in vivo.

**Results:**

MRL inhibited osteoclast differentiation and the associated bone resorption, by significantly decreasing osteoclastic gene expression. Mechanistically, MRL inhibited the phosphorylation of ERK by suppressing the mitochondrial function, thereby downregulating the nuclear factor of activated T cells 1 (NFATc1) signaling. LM22B-10 treatment further verified the targeted inhibition effect of MRL on ERK. Microscopic computed tomographic and histologic analyses of the tibial tissue sections indicated that ovariectomized mice had lower bone mass and higher expression of ERK compared with normal controls. However, MRL treatment significantly reversed these effects, indicating the anti-osteoporosis effect of MRL.

**Conclusion:**

We report for the first time that MRL inhibits ERK signaling by suppressing mitochondrial function, thereby ameliorating ovariectomy-induced osteoporosis. Our findings can provide a basis for the development of a novel therapeutic strategy for osteoporosis.

**Supplementary Information:**

The online version contains supplementary material available at 10.1186/s12967-023-04706-2.

## Background

The incidence of osteoporosis-the most common bone disease-is rapidly increasing worldwide due to increased life span and an aging population World Health Organization has recognized osteoporosis as a universal health problem. The disease mainly affects postmenopausal women, and its prevalence ranges from 3.2% in the 40–49 age group to19.2% in > 50 age group [1]. Osteoporosis is characterized by decreased bone density, damaged bone microstructure [2], increased bone fragility, and vulnerability to fracture [3]. Fractures caused by osteoporosis often occur in the centrum, hip, forearm distal, and proximal humerus, leading to disability and death in affected patients [4].

Bone health is regulated by tightly controlled homeostatic mechanisms. Osteoporosis mainly occurs due to the disruption of bone homeostasis, which depends on the balance of bone resorption by osteoclasts and bone formation by osteoblasts [2, 5]. Osteoclasts are the specialized multinucleated cells derived from the mononuclear macrophage lineage of hematopoietic cells in the bone marrow [6]. They can be activated by various pathological factors [7], such as chronic drug use, immobilization, malnutrition, and chronic inflammation [8, 9]. The abnormal activation of osteoclasts accelerates bone resorption and leads to osteoporosis and related diseases, such as Alzheimer’s disease, diabetes, and cancer [10, 11]. Therefore, inhibiting the formation and activity of osteoclasts can be a potential strategy for treating osteoporosis.

Oxidative stress is one of the critical factors inducing osteoporosis, and an increasing number of oxidative stress biomarkers are being detected in patients with osteoporosis [12, 13]. The accumulation of reactive oxygen species (ROS) promotes osteoclast differentiation and leads to hyper resorption of the bone [14–16]. Moreover, antioxidant treatment alleviates the symptoms of osteoporosis [17]. Therefore, ROS are involved in osteoclast differentiation, and attenuating ROS production could be a feasible strategy for preventing or treating osteoporosis. Mitochondria is the primary source of ROS in the cell [13]. Excessive mitochondrial ROS (mtROS) production results from the anomalous electron escapes from the electron transport chain, which disrupt normal signaling pathways and lead to abnormal osteoclast formation and differentiation [18, 19]. Excess intracellular ROS concentrations stimulate receptor activator of nuclear factor-κB ligand (RANKL) and enhance osteoclastogenesis [20]. Therefore, inhibiting mitochondrial ROS production may be an effective approach to suppress osteoclast activation and ameliorate osteoporosis.

Bone marrow-derived mononuclear pre-osteoclasts, fuse to form multinuclear osteoclasts [10]. Two important receptors expressed on the cell surface of pre-osteoclasts are colony stimulating factor-1 receptor (c-Fms) and RANK [6, 10]. Macrophage colony-stimulating factor (M-CSF) binds to c-Fms to promote survival, proliferation, and commitment of osteoclast precursor cells [6, 8]. The binding of RANK to RANKL is crucial for inducing osteoclast differentiation [7, 10]. Subsequently, TNF receptor-associated factor 6 (TRAF6) is recruited to the intracellular domain of RANK [9]. The activated TRAF6 initiates several downstream pathways, such as mitogen-activated protein kinase (MAPK) and nuclear factor-κB (NF-κB) pathways [11, 21]. These pathways are critical for osteoclast formation, differentiation, and activation [10]. Nuclear factor of activated T cells (NFATc1), master transcription factor in osteoclastogenesis, is induced because of the activated of these pathways [10]. Eventually, NFATc1 induces the expression of genes encoding osteoclast-relevant proteins, including tartrate-resistant acid phosphatase (TRAP), cathepsin K (CTSK), ATPase H^+^ transporting v0 subunit d2 (Atp6v0d2) [6, 22], matrix metalloproteinase (Mmp9) [6] and dendritic cell-specific transmembrane protein (Dcstamp) [10], which promote osteoclast formation and lead to bone resorption.

In addition, the MAPK signaling pathway is critical in osteoclast differentiation [23, 24]. This signaling pathway is linked to bone metabolism and is involved in osteoclast proliferation and differentiation [25, 26]. Moreover, the metabolism and function of mitochondria are tightly regulated by the MAPK signaling pathway. Its activation subsequently leads to the formation of mitochondria permeability transition pores and the ensuing dysfunction of mitochondria [27]. Moreover, MAPK activity regulates mitochondria-mediated pathways associated with survival and death [24]. However, the influence of MAPK-related mitochondrial function on osteoclast differentiation and osteoporosis remains unclear.

Several therapeutic strategies are available for osteoporosis. The most commonly used medications include calcium and vitamin D supplements (improve bone health), bisphosphonates and zoledronic acid (inhibit osteoclast activity) [8], and calcitonin (suppress bone resorption) [28]. However, these drugs have multiple side effects, such as epigastric discomfort, constipation, gastric and duodenal ulcers, reflux esophagitis, and breast cancer [29, 30]. Therefore, it is urgent to develop effective anti-osteoporosis drugs with minimal side effects.

Natural compounds derived from animals, plants, and microorganisms have unique pharmacological or biological activities [31]. These compounds and their metabolites are the potential sources of novel drugs for treating several diseases including osteoporosis [9]. Myrislignan (MRL) is a natural compound derived from *Myristica fragrans* (nutmeg) with extensive pharmacological activities [32, 33]. MRL induced surface shrinkage and mitochondrial damage in *Toxoplasma gondii*, a parasitic protozoan, eventually leading to its death [32]. In addition, MRL has anti-inflammatory and anti-cancer properties [32, 33]. However, the effect of MRL on osteoclasts activity remains unexplored. In this study, MRL showed anti-osteoclast activity and inhibited mitochondrial function in vitro. The anti-osteoclast effect of MRL was further verified in an ovariectomy (OVX) mouse model. In addition, the mechanisms by which MRL inhibits osteoclasts were explored, including the role of the MAPK/ERK signaling pathway and mitochondrial function.

## Methods

### Ethics

The Animal Center of Guangxi Medical University (Nanning, Guangxi, China) provided the C57BL/6 J mice. All animal experiments were performed with the approval of the Guangxi Medical University Ethics Committee (approval number: 202204009).

### Reagents and supplies

MRL was purchased from Chengdu Must Bio-Technology Co., Ltd (Chengdu, Sichuan, China). The compound had a purity of 99.24% and a molecular weight of 374.43 kDa. MRL was dissolved in dimethyl sulfoxide (DMSO) to prepare a 100 mM stock solution. The stock solution was diluted with minimum essential medium alpha modification (α-MEM) to prepare a 1 mM working solution. Lm22B-10 (LM; 99.72% pure; MW: 485.01 kDa) was bought from MedChemExpress (Shanghai, China). LM was dissolved in DMSO to prepare a 50 mM stock solution and then diluted with α-MEM to prepare a 500 μM working solution. α-MEM and fetal bovine serum (FBS) were procured from Gibco-Technology (Thermo Fisher Institute of Biotechnology, MD, United States). Recombinant mouse M-CSF and RANKL were supplied by the R&D Biotechnology Company (Minneapolis, MN, United States), and penicillin/streptomycin was procured from Thermo Fisher Scientific (Victoria, Australia). MedChemExpress provided cell counting kit-8 (CCK-8) and estrogen. Rever Aid RT Kit, MtROS red, and tetramethyl rhodamine methyl ester (TMRM) were provided by Thermo Fisher Scientific. Rhodamine-marked phalloidin and 4′6-diamidino-2-phenylindole (DAPI) were procured from Sigma-Aldrich (St. Louis, MO, United States). The prestained protein markers for western blotting was obtained from Thermo Fisher Scientific (Shanghai, China). Primary antibodies against NFATc1 (#sc-7294) and CTSK (#sc-48353) were provided by Santa Cruz Biotechnology (Dallas, CA, United States). Antibodies against Atp6v0d2 (#ab236375) and c-FOS (#ab134122) were procured from Abcam (Cambridge, England). Cell Signaling Technology (Danvers, MA, United States) supplied antibodies against phospho-NF-κB p65 (#3033), NF-κB p65 (#8242), IκBα (#4814), β-actin (#4970), p-ERK (#4370), ERK (#4695), p-JNK (#4668), JNK (#9252), p-P38 (#4511), P38 (#8690) and anti-mouse and anti-rabbit secondary antibodies.

### Extraction and culture of primary bone marrow macrophages (BMMs)

The femur and tibia of 4–6-weeks-old C57BL/6 J mice were separated. Bone marrow cells were harvested and resuspended into a complete medium containing 10% FBS, 1% penicillin–streptomycin, and 25 ng/ml M-CSF. The cells were cultured in a T-75 cell culture bottle at 37 ℃ under 95% O_2_ and 5% CO_2_ for 48 h.

### Cytotoxicity assay

BMMs were cultured in a CO_2_ incubator at 37 ℃ under 95% O_2_ and 5% CO_2_ for 36 h at a density of 7 × 10^3^ cells/well to ensure cell attachment. The supernatant was then replaced with different concentrations of drugs (MRL/LM) every 48 h. Next, 10 μL CCK-8 solution was added to the supernatant in each well, and the plate was incubated to culture for 2 h in a CO_2_ incubator. Optical density (OD) was measured using a Multimodule microplate meter (Berthold Technologies Gmbh & Co. KG, Baden-Wurttemberg, Germany). Finally, cell counts were determined from OD values to evaluate drug toxicity.

### BMM differentiation and TRAP staining

Approximately 7 × 10^3^ cells/well were seeded into 96-well plates and incubated for 36 h in a CO_2_ incubator to allow cell attachment. Next, the supernatants were replaced with different drug doses (MRL/LM) and RANKL (50 ng/ml). The procedure was repeated every 48 h for 1 week until mature multinuclear osteoclasts were formed in the control group. Next, the cells were fixed with 100 μL 4% of paraformaldehyde (PFA) for 2 h and then gently cleaned with phosphate-buffered saline (PBS) twice. Afterward, 70 μL TRAP dye liquor was added into each well for 15 min at 37 ℃to stain mature osteoclasts. Finally, plates were dried, and high-resolution images were obtained using Cytation 5 (Bio Tek Instruments Inc., Winooski, VT, USA). Osteoclasts with three or more nuclei were counted, and the ImageJ v1.53 software was used for quantitative calculations.

### Podosome belt formation and immunofluorescence experiment

We examined whether MRL impairs podosome belt formation and F-actin-mediated cytoskeletal structure. For this purpose, we seeded 7 × 10^3^ BMMs into 96-well plates and cultured them with different concentrations of MRL and 50 ng/ml RANKL for 7 days until mature osteoclasts attained a specific shape. The cells were then fixed with 4% PFA for 2 h and gently cleaned with PBS twice. Later, cells were treated with 0.1% Triton X-PBS at room temperature for 10 min and then PBS containing 3% bovine serum albumin (BSA) for 60 min. After being washed with PBS containing 0.2% BSA twice gently, cells were incubated with 1:200 rhodamine-phalloidin for 2 h in the dark. Cells were again successively washed with PBS and 0.2% BSA twice and stained with 1:100 DAPI for approximately 10 min in the dark. Finally, cells were gently rinsed with PBS twice, plates were dried, and full-hole fluorescent images were obtained using Cytation 5.

### Bone resorption assay

Osteoclasts are the only cells with bone resorption function in vivo; therefore, we used bovine bone slices to examine the resorptive function of osteoclasts in vitro. Bovine bone slices were dried using UV irradiation for about 40 min and serial numbers were marked on the back of dried slices. The pieces were then sequentially processed with absolute ethyl alcohol, PBS, and α-MEM for 48 h. Then, the slices were placed in 96-well plates with the numbered side facing down. Next, approximately 7 × 10^3^ BMMs were seeded on the bone slice in each well and incubated for 48 h to ensure attachment. Adherent cells were treated with RANKL for 4 days, to obtaining small osteoclasts. The supernatants were replaced with different concentrations of MRL containing RANKL for 2 days until control BMMs differentiated into mature multinuclear osteoclasts. Similar protocols were followed in only bovine bone slice or only TRAP stain controls.

Afterward, bovine bone slices in all groups were gently rinsed with PBS twice and fixed with 4% PFA for 1 h. Then, images were recorded using a scanning electron microscope (Manufacturer, City, Country, SU 8100, 3.0 kV). The area of the bone pit was recorded to determine the bone resorption capacity of osteoclasts. In the TRAP staining control group, cells were fixed using 4% PFA for 1 h and then gently rinsed with PBS twice. TRAP dye was added, and all images were recorded using Cytation 5. Mature osteoclasts were stained red, and the cells having three or more nuclei were counted for quantitative analysis.

### Quantitative reverse transcription-polymerase chain reaction (qRT-PCR)

Quantitative RT-PCR was performed to examine the mRNA expression of osteoclast marker genes. Approximately 1.5 × 10^5^ BMMs were seeded into 6-well plates and incubated for 36 h for cell attachment. The supernatant was then replaced with a fresh medium containing different concentrations of drugs (MRL/LM) and RANKL once in 2 days. When mature multinuclear osteoclasts were formed, TRIzol (Thermo Fisher Scientific) was added for 1 h to lyse them. Further, chloroform, isopropyl alcohol, and 75% ethyl alcohol were sequentially added to the lysis buffer for RNA extraction. The extracted RNA was reverse transcribed into complementary DNA (cDNA) using reverse transcription kits (Manufacturer, City, Country). The concentration of cDNA was measured and qRT-PCR was conducted using SYBR Green (Thermo Fisher Scientific) and RNA-specific primers. The reverse transcriptional conditions were 42 ℃ for 60 min, then 95 ℃ for 5 min, and finally 4 ℃ for 15 min, followed by amplification on a gradient PCR amplification instrument at 94 ℃ for 15 s, 58 ℃ for 25 s, and 72 ℃ for 25 s. All target gene expressions were quantified using the 2^−ΔΔ Ct^ method. All primer sequences are listed in Table 1.

**Table 1:** The primers used in this research

Gene	Forward primer (5′–3′)	Reverse primer (5′–3′)
*Nfatc1*	GGTGCTGTCTGGCCATAACT	GAAACGCTGGTACTGGCTTC
*Ctsk*	AGGCGGCTATATGACCACTG	TCTTCAGGGCTTTCTCGTTC
*Atp6v0d2*	GTGAGACCTTGGAAGACCTGAA	GAGAAATGTGCTCAGGGGCT
*Mmp9*	GAAGGCAAACCCTGTGTGTT	AGAGTACTGCTTGCCCAGGA
*Dcstamp*	GGAACCTAAGCGGAACTTAGACA	GCTAGGGCTTCGTGGAAACA
*Acp5*	ACGGCTACTTGCGGTTTCA	TCCTTGGGAGGCTGGTCTT
*Fos*	TACTACCATTCCCCAGCCGA	GCTGTCACCGTGGGGATAAA

### Mitochondria functional assays

BMMs were stimulated with 50 ng/ml RANKL and 30 μM MRL in 96-well plates for 3 days. Then, the spent medium was replaced and cells were washed with PBS. MitoSOX RED (5 μM) was added to each well, and plates were incubated for 10 min at 37 ℃ in a CO_2_ incubator protected from light. Finally, full-bore photographs were obtained using Cytation 5 (Bio Tek Instruments Inc.) in the dark. In addition, BMMs were similarly incubated for 7 days and then used to measure the membrane potential. The cells were gently washed using PBS and 100 nM TMRM was added into each well followed by incubation for 30 min at 37 ℃. Finally, the plates were kept in the dark, and images were recorded using Cytation 5. The average fluorescence value was determined using the ImageJ v1.53 software.

### Western blotting

Approximately 5 × 10^5^ BMMs were seeded to extract osteoclast-related short-term-acting proteins, and 1.5 × 10^5^ BMMs were seeded to obtain osteoclast-relevant long-term-acting proteins. BMMs were cultured with different concentrations of drugs (MRL/LM) and RANKL for 7 days to form mature osteoclasts and extract long-term-acting proteins. In contrast, BMMs were separately incubated with drugs (MRL/LM) and RANKL for 1 h each to isolate short-term-acting proteins. Next, BMMs were treated with RIPA lysis buffer including 1% phosphatase inhibitors, 1% protease inhibitor, and 1% phenylmethanesulfonyl fluoride for protein extraction. Finally, the extracted proteins were stored at − 80 ℃ till further use.

Further, the protein sample was mixed with a loading buffer and separated using sodium dodecyl sulfate–polyacrylamide gel electrophoresis. And the separated proteins were transferred onto the nitrocellulose membrane. The blotted membrane was blocked using skimmed milk at room temperature for 1 h to block unoccupied sites. The blocked membrane was rinsed with tris buffered saline containing tween (TBST) thrice and incubated overnight with the primary antibody at 4 ℃. The membrane was again washed thrice with TBST and incubated with a secondary antibody for 1 h at room temperature. Finally, protein bands were analyzed using ImageQuant LAS-4000 (GE Healthcare, Chicago, Illinois, USA).

### Establishment of OVX-induced osteoporosis mouse model

All animal experiments were carried out with the approval of the Guangxi Medical University Ethics Committee, including constructing an OVX-induced osteoporosis mouse model. Thirty 11-weeks-old female C57BL6/J mice were randomly divided into five groups: sham operation + vehicle group (Sham group), OVX + vehicle group (OVX group), OVX + 100 ng/kg E_2_ group (E_2_ group), OVX + 15 mg/kg MRL group (low-concentration group), and OVX + 30 mg/kg MRL group (high-concentration group). The mice were anesthetized using tribromoethanol (200 μL/10 g body weight), and mice from four groups (except for the Sham group) were ovariectomized to induce osteoporosis. Berberine was administered for 1 week, and mice were treated with group-specific reagents for 6 weeks. The mice from the Sham and OVX groups were intraperitoneally injected with normal saline containing 4% DMSO. Mice from the E_2_ group were intraperitoneally injected with 100 ng/kg E_2_. Low- and high-concentration groups of mice were administered 15 mg/kg and 30 mg/kg MRL respectively. Finally, all mice were sacrificed, and the tibias were removed for histological and microscopic-computed tomography (micro-CT) analysis.

### Statistical analysis

All the experiments were performed at least in triplicate. The data are presented as mean ± standard deviation or images. Student’s t-test or ANOVA was used for quantitative analysis, and *p < 0.05 was considered statistically significant unless otherwise stated.

## Results

### MRL inhibits RANKL-induced osteoclastogenesis in vitro

**Fig. 1:**
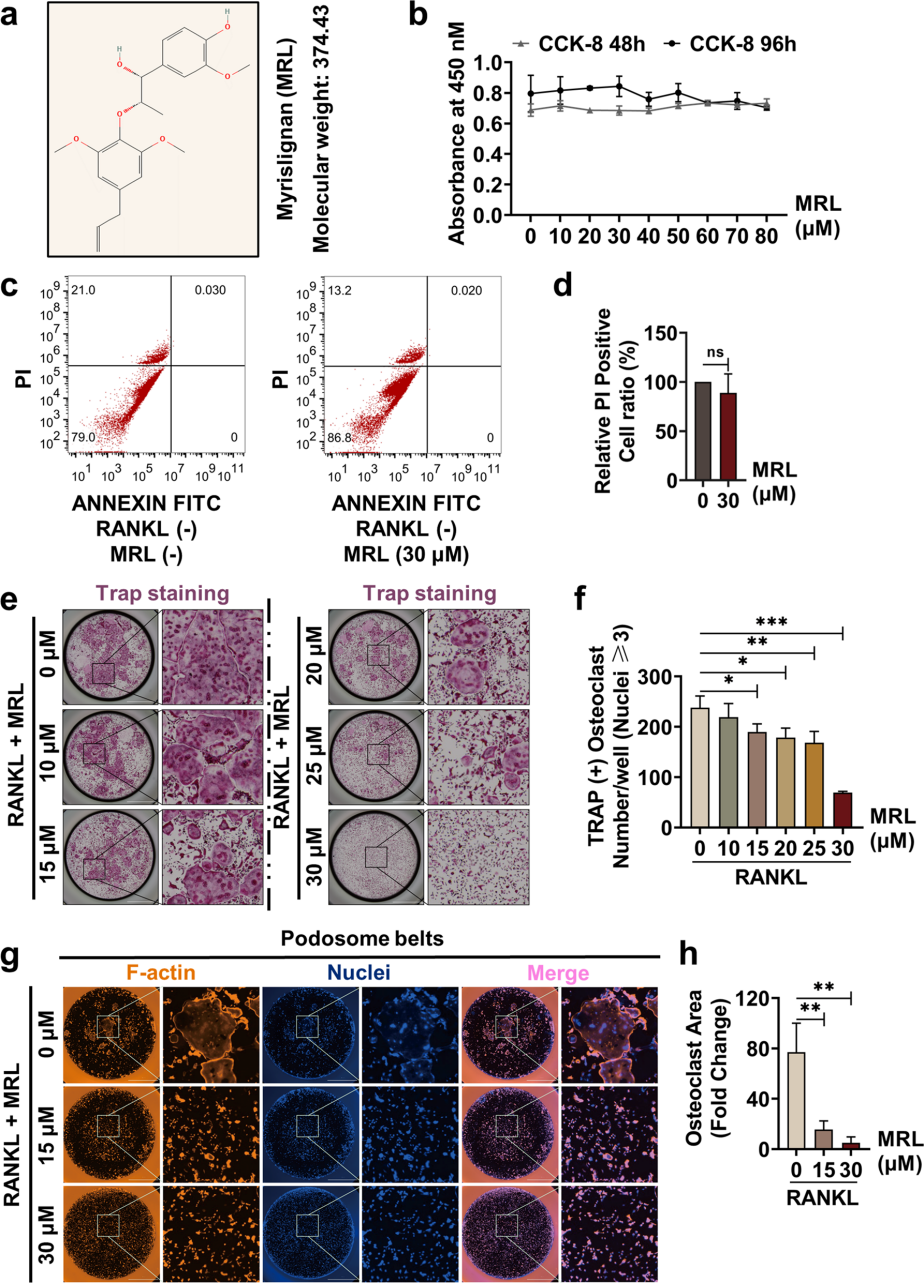
MRL inhibits RANKL-induced osteoclastogenesis in vitro. **a** The chemical structure and molecular formula of MRL. **b** BMMs cell proliferation was detected by CCK-8 assay after treatment with different concentrations of MRL for 48 and 96 h (n = 3 for each group). **c** Flow cytometric analysis of PI positive cell after BMMs were treated with MRL for 48 h. **d** Quantification of relative PI positive cell ratio of flow cytometric (n = 3 for each group). **e** Representative images of TRAP staining showed that MRL inhibited osteoclast formation in a dose-dependent manner after stimulation with 50 ng/ml RANKL for 7 days. Scale bar = 2000 μm. **f** Quantitative data of TRAP-positive osteoclasts per well was shown (n = 3 for each group). **g** Representative images of podosome belts in osteoclasts treated with different concentrations of MRL. Scale bar = 2,000 μm. **h** Quantitative data of mean F-actin ring area per well (n = 3 for each group). All the data were expressed as mean ± SD. *p < 0.05, **p < 0.01 and ***p < 0.001

Figure 1a show the chemical structure and molecular formula of MRL. The results of CCK-8 assays revealed that the activity of BMMs was not impaired even after the treatment with 80 μM MRL for 48 and 96 h (Fig. 1b). Next, t we conducted flow cytometric analysis and found that MRL did not affect the apoptosis ratio of BMMs (Fig. 1c, d). Then, we determined the effect of MRL on the differentiation of BMMs to osteoclasts. BMMs were seeded into 96-well plates and incubated with 50 ng/ml RANKL and different concentrations of MRL for 7 days. Then, BMMs were fixed with PFA and stained with the TRAP dye after the formation of mature multinucleated osteoclasts. Osteoclast differentiation was inhibited with the increasing concentration of MRL (Fig. 1e, f). After being stimulated with RANKL for 7 days, mature osteoclasts were stained with rhodamine-conjugated phalloidin. Intact podosome belts associated with actin cytoskeleton formation and morphology were red, and cell nuclei were blue. Treatment with both high and low concentrations of MRL markedly prevented F-actin ring formation even with 50 ng/ml RANKL stimulation (Fig. 1g, h). Overall, these results demonstrate that MRL had an inhibitory effect on osteoclast differentiation.

### MRL impairs the bone resorption function and inhibits the expression of osteoclast-related genes in mature osteoclasts in vitro

**Fig. 2:**
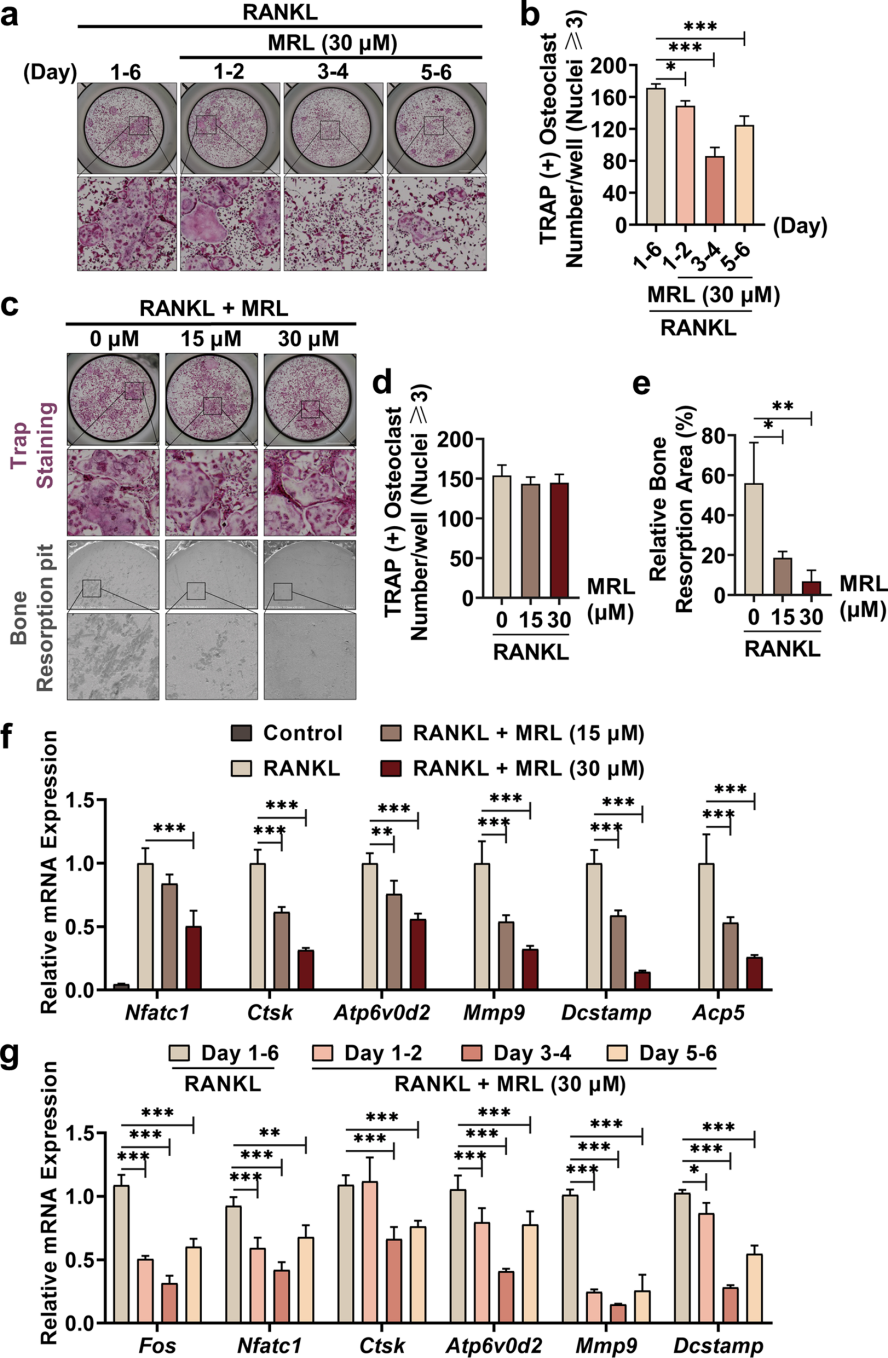
MRL damages the bone resorption function and inhibits the expression of osteoclast-related genes in vitro. **a** Representative images of TRAP staining showed that MRL of 30 μM inhibited osteoclast formation in a time-dependent manner after stimulation with 50 ng/ml RANKL. Scale bar = 2000 μm. **b** Quantitative data of TRAP-positive osteoclasts per well treated with MRL of 30 μM at different periods (n = 3 for each group). **c** Representative images of TRAP staining, which was as control. Scale bar = 2000 μm. And representative images of bovine bone slices seeded into the same number of mature osteoclasts stimulated by 50 ng/ml RANKL and MRL. The area of the bone pit showed the capacity for bone resorption. Scale bar = 1,000 μm. **d** Quantitative data of TRAP-positive osteoclasts per well was shown (n = 3 for each group). **e** Quantitative data of relative bone resorption area per well (n = 3 for each group). **f** qRT-PCR showed the levels of relative mRNA expression of osteoclast-related genes, including *Nfatc1*, *Ctsk*, *Atp6v0d2*, *Mmp9*, *Dcstamp*, and *Acp5*, which were detected in the presence of different concentrations of MRL and 50 ng/ml RANKL (n = 3 for each group). **g** qRT-PCR showed the levels of relative mRNA expression of osteoclast-specific genes, covering *Fos*, *Nfatc1*, *Ctsk*, *Atp6v0d2*, *Mmp9*, and *Dcstamp*, which were detected in the presence of 30 μM MRL and 50 ng/ml RANKL at different stages of osteoclast differentiation (n = 3 for each group). All the data were expressed as mean ± SD. *p < 0.05, **p < 0.01 and ***p < 0.001

We treated BMMs with 30 μM of MRL for varying durations and RANKL. MRL inhibited osteoclastogenesis during in the middle stage (day 3–4) of osteoclast differentiation because a minimum number of TRAP-positive osteoclasts were counted at this stage (Fig. 2a, b). Further, treatment with a that high concentration of MRL decreased the area of the bone pit formed by osteoclasts in bovine bone slices compared with only RANKL treatment. Notably, the number and morphology of TRAP-positive osteoclasts were similar in both cases (Fig. 2c–e).

BMMs were treated with different concentrations of MRL and RANKL to induce their differentiation into mature multinuclear osteoclasts. High concentrations of MRL inhibited the relative expression of osteoclast marker genes compared with the RANKL. These genes included *Nfatc1*, *Ctsk*, *Atp6v0d2*, *Mmp9*, *Dcstamp*, and acid phosphatase (*Acp5*) (Fig. 2f). We also extracted mRNA at different time points of osteoclast differentiation. MRL mainly suppressed osteoclast-relevant gene expression in the middle stage of osteoclast differentiation, including the expression of proto-oncogene c-Fos (*Fos*), *Nfatc1*, *Ctsk*, *Atp6v0d2*, *Mmp9*, and *Dcstamp* (Fig. 2g).

Long-term-acting proteins were extracted after the formation of mature multinuclear osteoclasts, when BMMs treated with 50 ng/ml RANKL for 7 days and 30 μM MRL for 0, 1, 3, and 5 days. The expressions of osteoclast-related proteins, including c-FOS, NFATc1, CTSK, and Atp6v0d2, were inhibited by MRL (Additional file 1: Fig. S1).

### MRL inhibits the function of mitochondria and mitochondrial-induced osteoclast differentiation

**Fig. 3:**
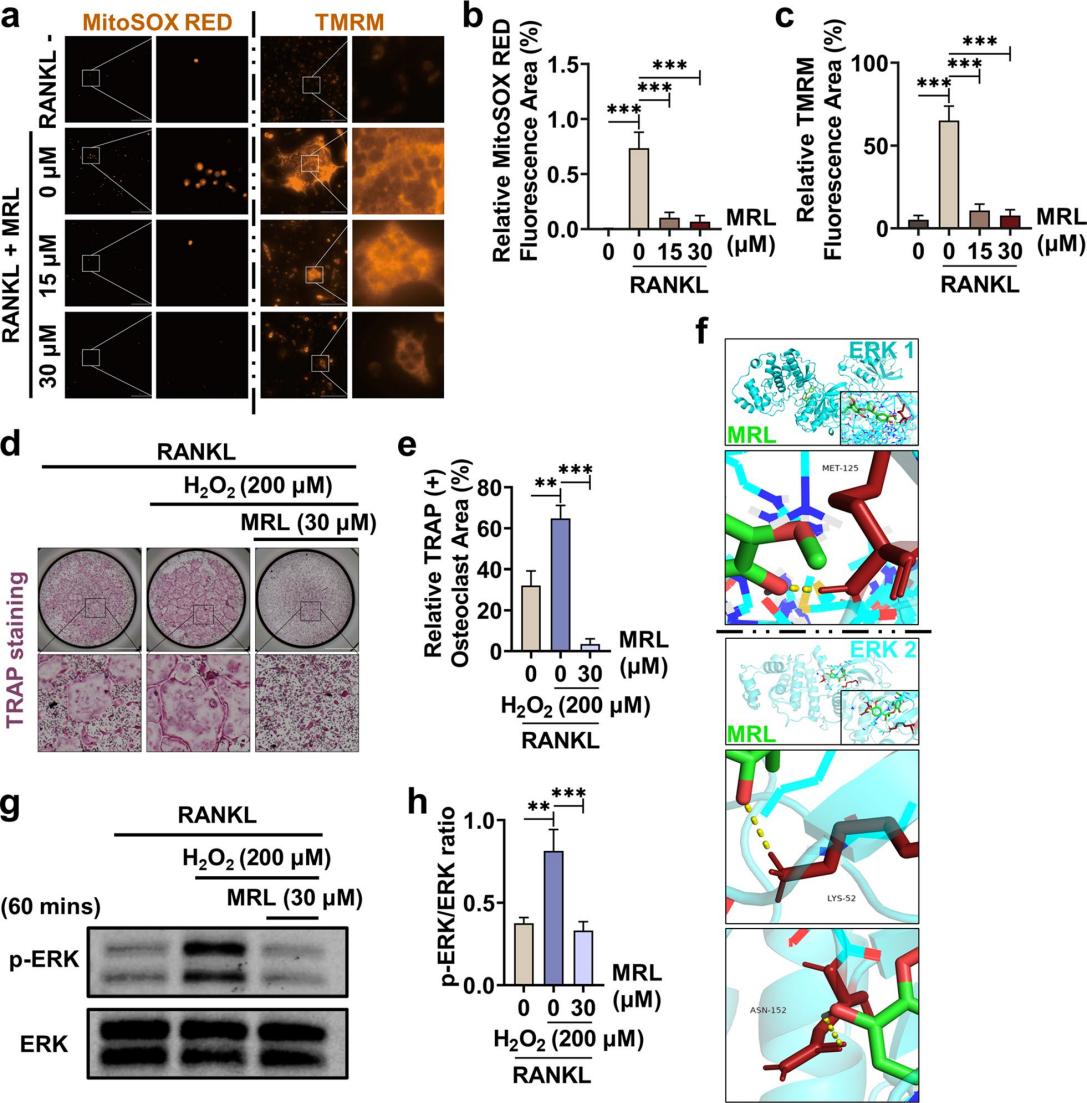
MRL inhibits the function of mitochondria and mitochondrial-induced osteoclast differentiation. **a** Representative images of MitoSOX RED staining and TMRM staining. After BMMs were treated with 50 ng/ml RANKL and 30 μM MRL for 48 h, BMMs were stained with MitoSOX RED indicator for 10 min at 37℃ temperatures. Scale bar = 300 μm. BMMs were treated the same way for 7 days and then stained with TMRM reagent for 30 min to analyze osteoclast mitochondrial function. Scale bar = 100 μm. **b** Quantitative data of relative MitoSOX RED fluorescence area was displayed (n = 3 for each group). **c** Quantitative data of relative TMRM fluorescence area was shown (n = 3 for each group). **d** Representative images of TRAP staining of BMMs treated with MRL of 30 μM and H_2_O_2_ of 200 μM in 50 ng/ml RANKL. Scale bar = 2,000 μm. **e** Quantitative data of relative TRAP-positive osteoclast area per well (n = 3 for each group). **f** There was one hydrogen bond (yellow) between MRL (green) and the amino acid residues MET-125 (red) of ERK 1. While two hydrogen bonds (yellow) were formed between MRL (green) and the amino acid residues LYS-52 and ASN-152 (red) of ERK 2. **g** After BMMs were treated with 200 μM H_2_O_2_ and 30 μM MRL for 60 min in the presence of 50 ng/ml RANKL, the expression of ERK was detected by Western Blotting. **h** Quantitative data of band intensity ratios of phosphorylated protein p-ERK relative to total protein ERK (n = 3 for each group). All the data were expressed as mean ± SD. *p < 0.05, **p < 0.01 and ***p < 0.001

Mature osteoclasts have high mitochondrial activity to provide more energy for promoting bone resorption. Furthermore, mitochondrial activation of the MAPK pathway plays an active role in osteoclast formation and differentiation. We performed MitoSOX RED and TMRM staining of BMMs to detect mitochondrial activity. The staining intensity of MitoSOX RED and TMRM increased in the RANKL group, whereas the staining intensity declined in the MRL group, indicating a significant decrease in mitochondrial activity after MRL treatment (Fig. 3a–c). Next, we added H_2_O_2_ to BMMs for increasing of ROS concentrations in vitro. Although the number of osteoclasts increased in the H_2_O_2_-treated group increased, osteoclast differentiation was significantly inhibited after MRL administration (Fig. 3d, e). Taken together, H_2_O_2_-mediated mitochondrial activity promoted osteoclast differentiation, and MRL treatment inhibited both mitochondrial activity and osteoclast differentiation.

Next, we performed molecular docking to examine the interaction between MRL and osteoclast-related proteins (Fig. 3f). We observed one hydrogen bond between MRL and MET -125 residue of extracellular signal-regulated kinase (ERK)1 and two bonds between MRL and LYS-52 and ASN-152 residues of ERK2. Therefore, a strong binding was observed between MRL and ERK. Moreover, phosphorylation of the ERK protein was activated by H_2_O_2_ but inhibited by MRL (Fig. 3g, h). Overall, mitochondria promoted osteoclast differentiation by increasing ERK phosphorylation, and MRL inhibited mitochondrial function and ERK phosphorylation to suppress osteoclast differentiation.

### MRL inhibits ERK in vitro

**Fig. 4:**
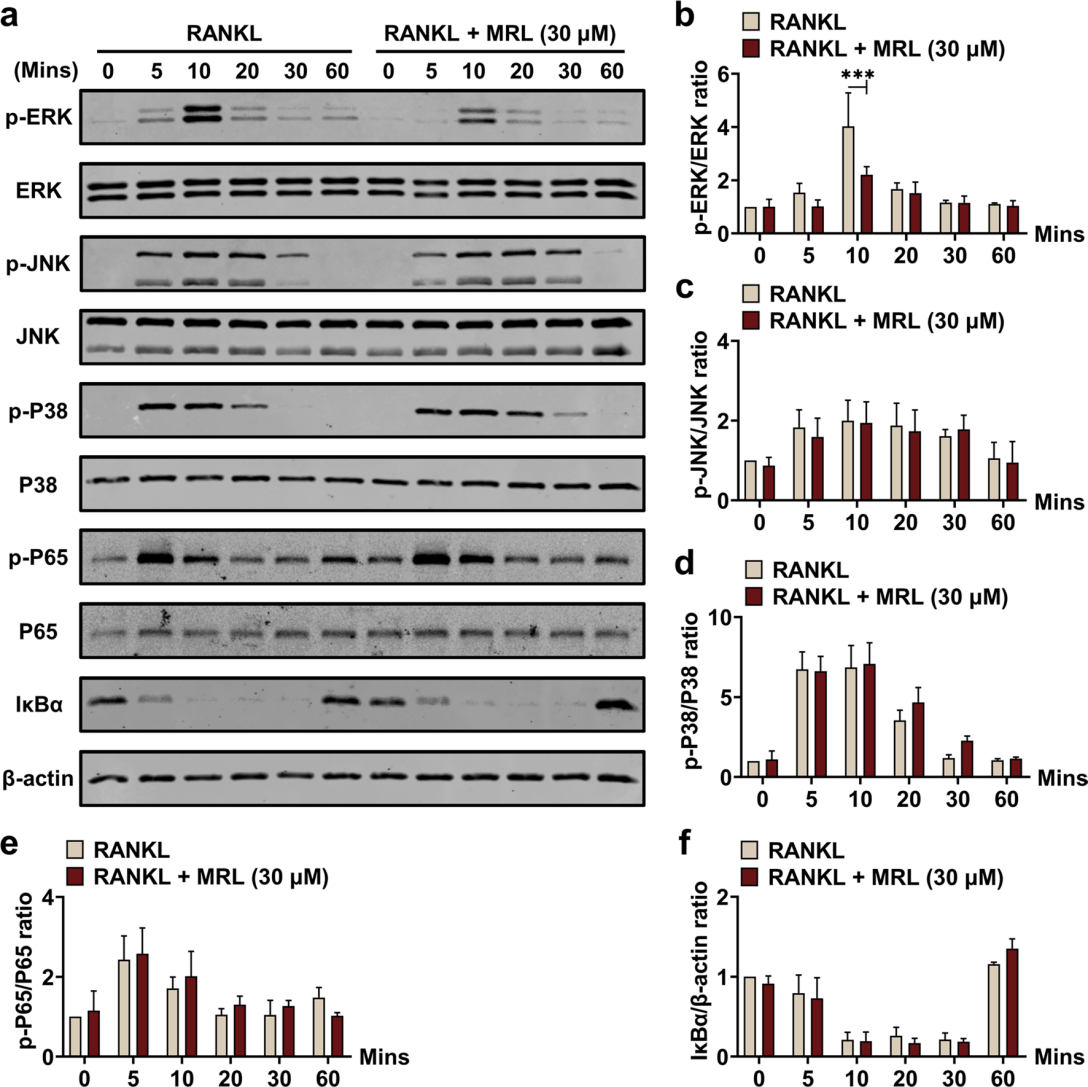
MRL has a restraining effect on protein ERK in vitro. **a** After BMMs being stimulated with 30 μM MRL for 60 min and 50 ng/ml RANKL for 0, 5, 10, 20, 30, and 60 min, the phosphorylation of osteoclast-related early-stage signaling protein ERK was inhibited markedly by MRL. Besides, MRL did not affect the expression of JNK and P38. And the phosphorylation of osteoclast-related early-stage signaling protein P65 and IκBα were not suppressed by MRL. **b**–**f** Quantitative data of band intensity ratios of phosphorylated protein p-ERK relative to total protein ERK. And quantitative data of band intensity ratios of phosphorylated protein p-JNK relative to total protein JNK and phosphorylated protein p-P38 relative to total protein P38. Quantitative data of band intensity ratios of phosphorylated protein p-P65 relative to total protein P65 and IκBα relative to β-actin (n = 3 for each group). All the data were expressed as mean ± SD. *p < 0.05, **p < 0.01 and ***p < 0.001

Short-term-acting proteins were extracted after osteoclasts were treated with 50 ng/ml RANKL for 60 min and MRL (0 or 30 μM) for 0, 5, 10, 20, 30, and 60 min. The MAPK pathway is a classical osteoclast differentiation pathway downstream of the RANK pathway. This pathway involves ERK, P38, and c-Jun N-terminal kinase (JNK), which are the early-stage osteoclast-related proteins. Therefore, we investigated the effect of MRL on the MAPK pathway. MRL inhibited the phosphorylation of ERK but not of JNK or P38 (Fig. 4a–d). Molecular docking and western blotting results implied that MRL specifically binds to ERK. However, MRL did not influence the short-term-acting proteins of osteoclast differentiation, including P65 and IκBα, which participate in the NF-κB pathway (Fig. 4e, f).

### MRL inhibits osteoclastogenesis by reducing phosphorylation of the ERK protein of the MAPK pathway in vitro

We added LM (a specific ERK agonist) to further examine the inhibition of ERK by MRL. The application of LM alone or in combination with MRL did not affect the proliferation of BMMs (Additional file 1: Fig. S2a, b). BMMs were treated with MRL, LM, or MRL + LM, and then stimulated with RANKL for 7 days until mature multinuclear osteoclasts were formed. TRAP staining results revealed an increase in the number of osteoclasts after LM treatment compared with the RANKL treatment for 7 days (Additional file 1: Fig. S2c, d). However, BMMs treated with MRL could not differentiate into mature multinucleated osteoclasts, and BMMs treated with a combination of MRL and LM were only partially differentiated.

**Fig. 5:**
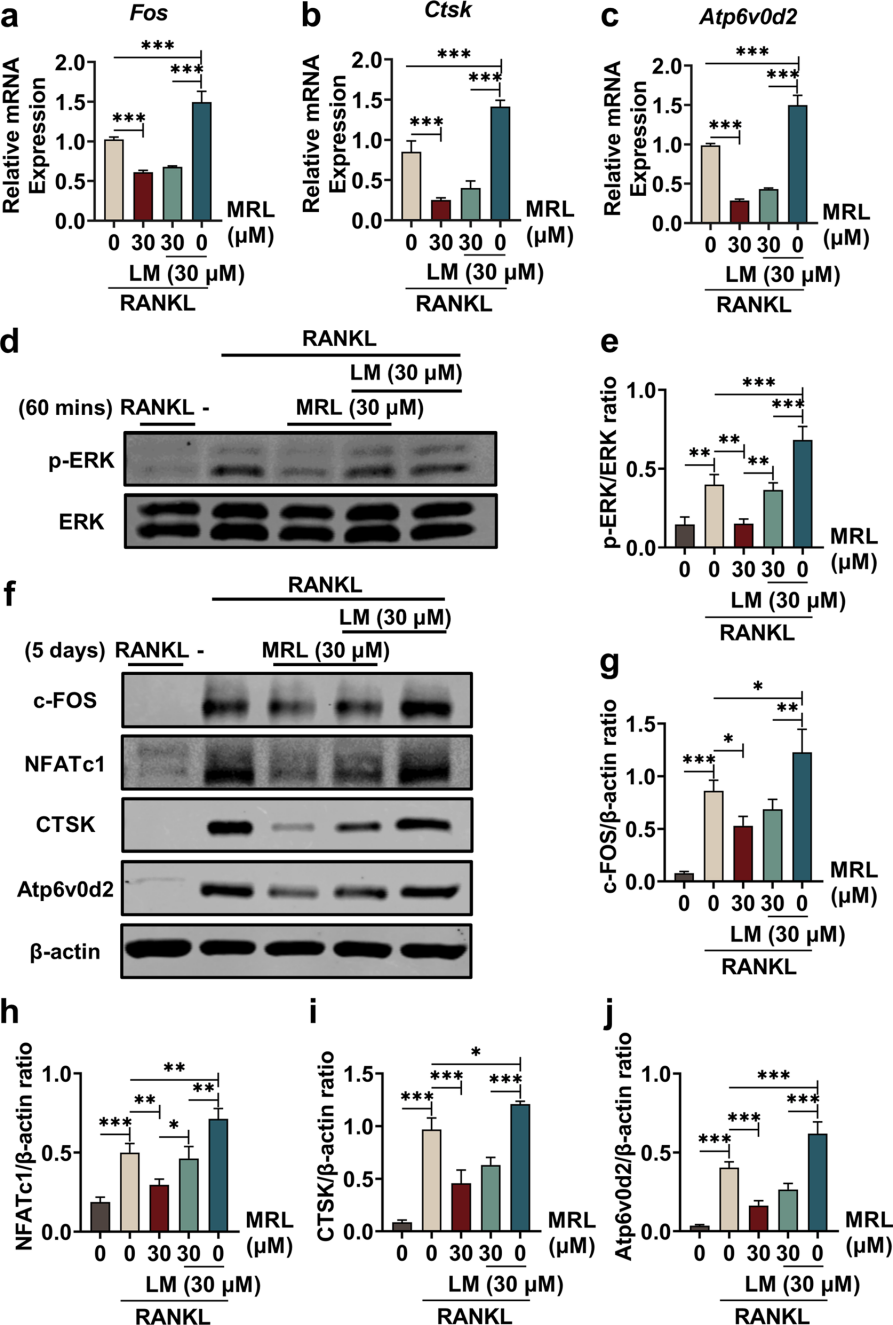
MRL inhibits osteoclastogenesis via reducing phosphorylation of ERK protein of MAPK pathway in vitro. **a**–**c** qRT-PCR showed the levels of relative mRNA expression of osteoclast-related genes, including *Fos*, *Ctsk*, and *Atp6v0d2*, which were detected when 30 μM MRL and 30 μM LM were applied separately or jointly (n = 3 for each group). **d** BMMs were stimulated with 50 ng/ml RANKL for 60 min, 30 μM MRL and 30 μM LM dividually or unitedly for 60 min to detect osteoclast-related protein ERK expression. Western Blotting showed that LM promoted corelative protein ERK expression while MRL restrained related ERK expression. **e** Quantitative data of band intensity ratios of phosphorylated protein p-ERK relative to total protein ERK (n = 3 for each group). **f** After BMMs were stimulated with 50 ng/ml RANKL for 5 days, 30 μM MRL and 30 μM LM were individually or unitedly used to stimulate osteoclast-related downstream protein c-FOS, NFATc1, CTSK, and Atp6v0d2 expression. Western Blotting showed the result. **g**–**j** Quantitative data of band intensity ratios of c-FOS, NFATc1, CTSK, and Atp6v0d2 relative to β-actin (n = 3 for each group). All the data were expressed as mean ± SD. *p < 0.05, **p < 0.01 and ***p < 0.001

The osteoclast-relevant genes, including Fos, Ctsk, and Atp6v0d2, were detected at the transcriptional level (Fig. 5a–c). The results of qRT-PCR also revealed that LM promoted the expression of osteoclast-related mRNAs, whereas MRL inhibited their expression. Long-term-acting proteins were extracted after BMMs were treated with MRL, LM, MRL + LM and stimulated with RANKL for 7 days until mature multinuclear osteoclasts were formed. Similarly, short-term-acting proteins were extracted after subjecting BMMs to similarly treatment for 60 min. LM promoted the expression of ERK, whereas MRL suppressed its expression. The phosphorylation level of ERK in the BMMs treated with combined LM and MRL treatment was the average of the levels obtained with LM and MRL treatment (Fig. 5d, e). A similar trend was observed in the expression of the downstream proteins, including c-FOS, NFATc1, CTSK, and Atp6v0d2 (Fig. 5f–j). Overall, these findings confirmed that MRL inhibited osteoclast function by modulating the ERK protein expression in vitro.

### MRL reduces bone loss in OVX mice in vivo

We established an OVX-induced osteoporosis mouse model to further confirm the therapeutic effect of MRL. The ovariectomized mice were divided into five groups: SHAM, OVX, 100 ng/kg E_2_, 15 μM MRL, and 30 μΜ MRL groups.

**Fig. 6:**
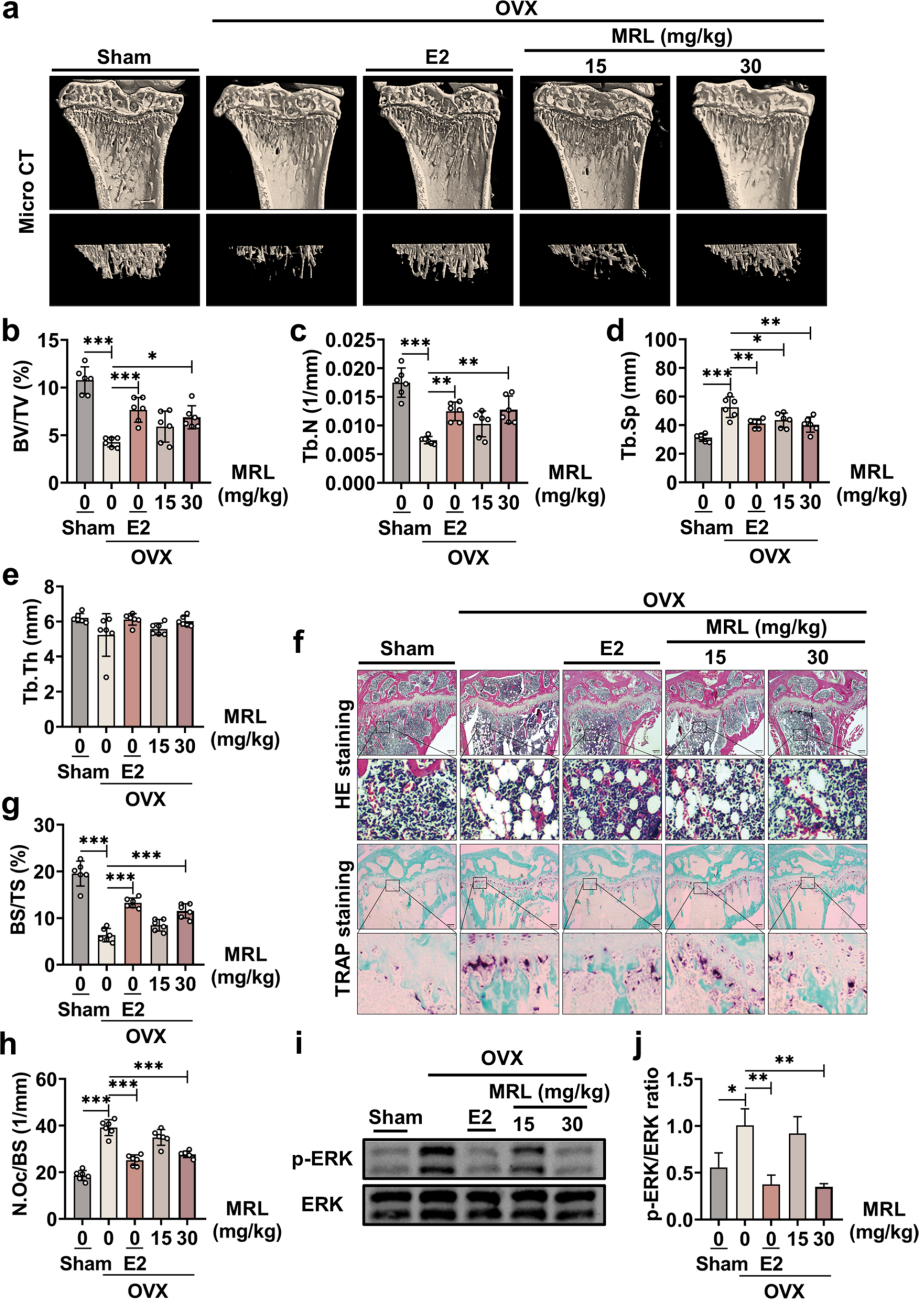
MRL reduces bone loss in ovariectomy mice in vivo. **a** Representative images of Micro CT that were reconstructed in three dimensions. The area scanned was the proximal tibia in indicated groups. **b**–**e** The relevant bone parameters analyzed included the following four: BV/TV, Tb.N, Tb.Sp, and Tb.Th (n = 6 for each group). **f** Representative images of histological analysis of tibia stained with HE. Scale bar = 300 μm. And representative images of histological analysis of the tibia stained with TRAP. Scale bar = 300 μm. **g** Quantitative data analysis of BS/TS (n = 6 for each group). **h** Quantitative data analysis of TRAP-positive cell area per trabecular surface (N.Oc/BS) (n = 6 for each group). **i** Representative images of Western Blotting showed the expression of p-ERK. **j** Quantitative data of band intensity ratio of p-ERK relative to ERK (n = 3 for each group). All the data were expressed as mean ± SD. *p < 0.05, **p < 0.01 and ***p < 0.001

The body weight increased in all five groups of mice, and no abnormal events occurred (Additional file 1: Fig. S3). The micro-CT results indicated a significant decrease in bone mass in the OVX group. In contrast, the bone mass significantly increased in the E_2_ and 30 μM MRL groups (Fig. 6a–e). HE and TRAP staining images also indicated similar observations, suggesting the therapeutic effect of MRL on osteoporosis (Fig. 6f–h).

**Fig. 7:**
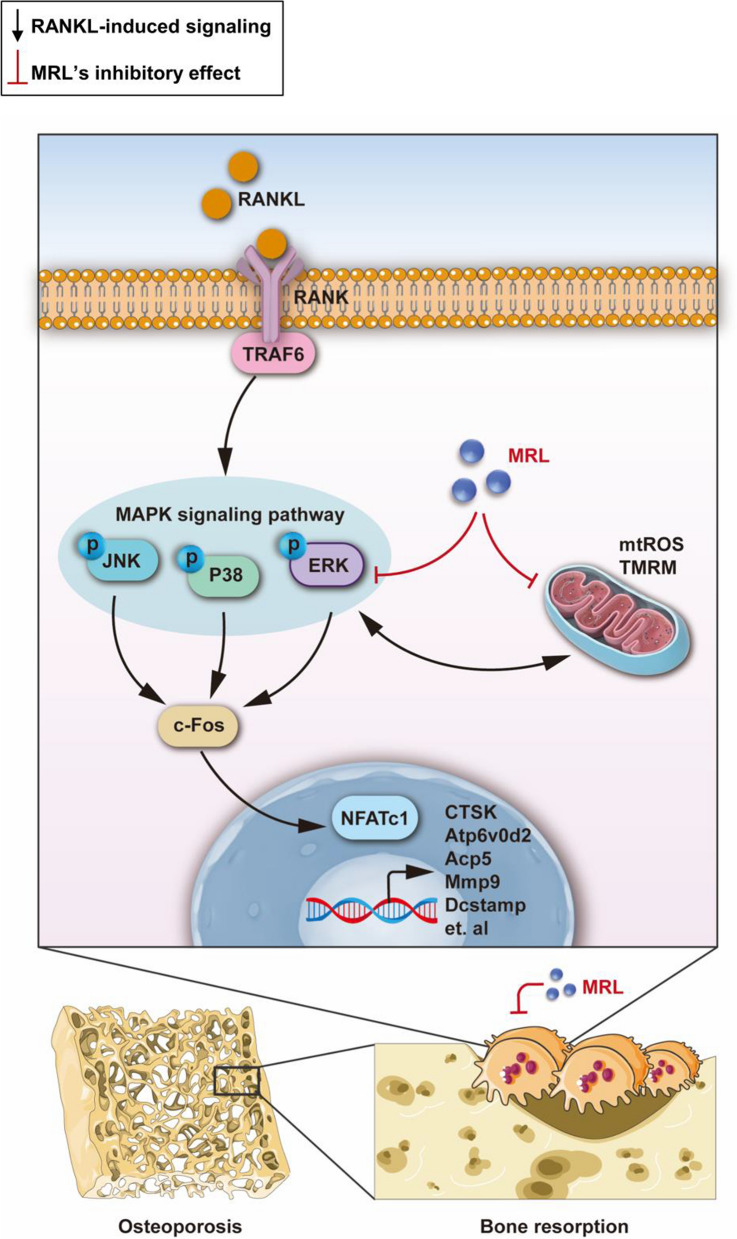
A schematic mechanism diagram showing the inhibitory effect of MRL on osteoclastogenesis. Our results reveal the inhibiting effect of MRL on osteoclast formation via suppressing mitochondrial function and ERK signaling for the first time. Besides, MRL partially reduces bone loss in mice after ovariectomy

MRL and ERK bound to each other in vitro; therefore, we extracted upper limb proteins from the five groups of mice to validate this finding in vivo. Phosphorylation of the ERK protein was significantly increased in the OVX group but decreased in the E_2_ and 30 μM MRL groups (Fig. 6i, j). Overall, these findings suggested that MRL inhibited ERK phosphorylation to suppress osteoclast differentiation and protect bone mass. Figure 7 presents the schematic mechanism of the inhibitory effect of MRL on osteoclastogenesis.

## Discussion

Osteoporosis is characterized by a disturbance in bone homeostasis, decline in bone mass, and degeneration of bone microstructure [34, 35]. The disease is closely regulated by osteoclasts and osteoblasts. Osteoblasts play a significant role in bone formation, whereas osteoclasts resorb bone and release the mineral matrix [36]. Osteoporosis occurs when the delicate balance of bone remodeling is disturbed [37]. The disease is mainly induced by the excessive activation of osteoclast differentiation [38, 39], resulting from altered RANK/RANKL signaling [40, 41] and other downstream pathways. In addition, ROS can induce osteoclast differentiation [42, 43]. The ROS-related mitochondrial activity is closely associated with osteoclast differentiation [44]. Therefore, the inhibition of osteoclast activity and mitochondrial function may lead to the amelioration of osteoporosis.

Several adverse effects are associated with the long-term use of drugs that inhibit osteoclast function and generation [8, 38]. For instance, anti-resorptive bisphosphonate drugs can effectively block the bone resorption function of osteoclasts but destroy the normal bone remodeling process causing mandibular osteonecrosis and atypical femur fractures [41, 45, 46]. Therefore, developing novel osteoporosis treatments with minimal side effects is urgently required. Natural products have the advantages of availability, low manufacturing cost, and lack of adverse effects [22, 38]. In this study, we found that MRL, a bioactive neolignane, inhibited the differentiation of osteoclasts by suppressing mitochondrial function, inhibiting phosphorylation of ERK, and activation of NFATc1. In addition, the anti-osteoclastic effects of MRL were verified in an OVX mouse model.

Osteoclasts are responsible for removing damaged bone tissue and initiating bone remodeling because of their bone-resorbing property [6, 41, 47]. The formation and differentiation of osteoclasts are strictly controlled under physiological conditions. However, various pathological factors could stimulate the formation of osteoclasts and lead to osteolytic bone disease [7, 41, 48]. In this study, MRL did not affect BMM proliferation and apoptosis but inhibited RANKL-induced osteoclast formation in a dose- and time-dependent manner. Specifically, MRL inhibited the middle stage of osteoclastogenesis. Furthermore, MRL reduced the actin-ring formation and resorption function of mature osteoclasts in a dose-dependent manner, impairing osteoclast skeleton formation.

Mitochondria are essential to fulfill high energy requirements of mature osteoclasts [13, 35, 49]. Mature multinuclear osteoclasts appear to increase mitochondria activity and ROS levels [50], and mitochondrial induced H_2_O_2_ production promotes osteoclastogenesis and bone resorption [51, 52]. Therefore, the relationship between mitochondria and osteoclastic differentiation is worth exploring. Here, MRL treatment decreased the intensity of MitoSOX RED and TMRM staining compared with the RANKL treatment, suggesting the inhibitory effect of MRL on mitochondrial function. In addition, the relative area of osteoclasts became more prominent with the addition of H_2_O_2_. In contrast, osteoclasts decreased in size after MRL treatment. Overall, MRL inhibited mitochondrial function and osteoclast differentiation.

RANKL and M-CSF are crucial cytokines that regulate osteoclast formation and differentiation [3, 38, 53]. The survival and proliferation of osteoclast precursors are closely associated with the binding of M-CSF and c-Fms [21, 54]. The RANKL/RANK interaction is indispensable for osteoclastogenesis [35, 38, 40]. This binding activates the downstream MAPK and NF-κB pathways to stimulate osteoclast formation [55]. The classical NF-κB signaling pathway involves the IκB kinase complex [56], which can degrade IκBα and result in transcription initiation [34, 47]. The MAPK pathway, involving ERK, JNK, and P38, promotes osteoclast formation and differentiation [53]. The ERK signaling pathway is crucial for the survival and differentiation of osteoclasts, whereas the P38 signaling pathway is indispensable for osteoclastogenesis but not for the fusion of bone marrow-derived pre-osteoclasts [54]. In the present study, MRL inhibited RANKL-induced osteoclast formation by suppressing ERK phosphorylation without affecting P65, IκBα, JNK, and P38. Molecular docking results and the application of LM (an ERK activator) to BMMs further confirmed the inhibitory effect of MRL on ERK.

The inactivation of ERK could reduce the membrane potential of mitochondria, suppressing mitochondrial function [57]. ERK signaling maintains mitochondrial homeostasis and avoids inflammatory stimulation to maintain mitochondrial function, thereby providing energy for osteoclastogenesis [44]. Moreover, mitochondria-mediated pro-survival and pro-death pathways are closely related to ERK signaling [4]. Therefore, ERK signaling is strongly linked with mitochondrial function. Here, we found that mitochondria-induced H_2_O_2_ promoted osteoclast differentiation with higher phosphorylation of ERK, and MRL treatment inhibited these effects.

The activation of the NF-κB or MAPK signaling pathway leads to the phosphorylation of [34, 58], which is a critical transcription factor for osteoclast differentiation [39]. Transcription factor c-Fos is vital for early induction of NFATc1 [41] a master regulator of osteoclast differentiation [41]. Embryonic stem cells lacking NFATc1 cannot respond to RANK stimulation and lose the ability to produce osteoclasts [39, 59]. Mice deficient in NFATc1 cannot degrade primary spongiosa due to increased bone mass, leading to calcified cartilage build-up and severe osteosclerosis [37, 60]. The promoter region of NFATc1 is activated by c-Fos, leading to enhanced transcription of NFATc1 during osteoclastogenesis [38] and its subsequent nuclear translocation. Afterward, multiple synergistic genes are upregulated by NFATc1 activation, including Mmp9, Atp6v0d2, Acp5, Dcstamp, and CTSK, which promote the formation of mature osteoclasts and bone resorption [34, 35, 56]. MMP-9 is indispensable for the migration and resorption function of mature osteoclasts [9, 34, 46]. Atp6v0d2 regulates H^+^ secretion and dissolves the crystalline hydroxyapatite and organic matrix [34, 61, 62]. CTSK is mainly responsible for degrading collagen and other matrix proteins [34]. CTSK-deficient mice show hyper-enhanced mineralization of bone tissue and abnormal bone remodeling [29, 30, 34]. In our study, MRL decreased the activity of NFATc1 by inhibiting the phosphorylation of c-Fos. Moreover, MRL inhibited the expression of MMP-9, Atp6v0d2, CTSK, and other osteoclast-relevant proteins, thereby inhibiting the bone resorption function of osteoclasts.

An OVX mouse model was constructed to validate whether MRL could inhibit the function of osteoclasts and osteoporosis in vivo. The bone loss feature in the mouse model was similar to that in postmenopausal women [22]. Micro-CT and histological findings indicated that MRL treatment reduced bone loss in mice. The MRL treated group had more bone trabeculae and fewer osteoclasts, showing a favorable therapeutic effect. In addition, western blotting results demonstrated the inhibitory effect of MRL on the ERK protein, consistent with the in vitro findings.

However, this study has some limitations that need to be addressed further. MRL can inhibit the activity of osteoclasts at different concentrations. However, bone remodeling results from the combined activity of osteoblasts and osteoclasts. Therefore, the mechanisms by which MRL modulates the activity of osteoblasts need to be further studied.

## Conclusion

MRL shows an anti-osteoclast activity both in BMMs and a mouse model. It inhibits mitochondrial function, ERK phosphorylation, and NFATc1 activation. The anti-osteoclastic activity of MRL can be exploited to develop a novel osteoporosis treatment with minimal side effects. Recently, some authors have reported that MRL induces redox imbalance and activates autophagy [32]. The MAPK/ERK pathway is also involved in redox imbalance and autophagy [63]. Therefore, inhibiting osteoclast activity and preventing osteoporosis may only be one of the effects of MRL on the MAPK/ERK pathway, and other outcomes of this inhibitory effect need to be explored further.

### Supplementary Information

**Additional file 1: Figure S1.** MRL inhibits RANKL-induced NFATc1 expression *in vitro*. (a) After being stimulated with 50 ng/ml RANKL for five days and 30 μM MRL for 0, 1, 3, and 5 days, the expression of osteoclast-related downstream signaling proteins was detected by Western Blotting. (b-e) Quantitative data of band intensity ratios of c-FOS, NFATc1, CTSK, and Atp6v0d2 relative to β-actin. **Figure S2.** LM has a negative toxic effect on BMMs. (a) BMMs cell proliferation was detected by CCK-8 assay after treatment with different concentrations of LM for 48 hours. (b) Cell proliferation of BMMs was detected by CCK-8 assay after being unitedly treated with 30 μM MRL and different concentrations of LM. (c) Representative images of TRAP staining showed that after stimulation with 50 ng/ml RANKL for seven days, osteoclast differentiation was activated by 30 μM LM but was suppressed by 30 μM MRL. (d) Quantitative data of TRAP-positive osteoclasts per well was shown. **Figure S3.** The weight of OVX-induced mice. (a) Verifying the successful construction of the OVX-induced model was done by measuring the body weight of the mice.

## Data Availability

The datasets used and/or analyzed during the current study are available from the corresponding author on reasonable request.

## Abbreviations

Acp5: Acid phosphatase 5
Atp6v0d2: ATPase H^+^ transporting v0 subunit d2
BMMs: Bone marrow macrophages
BSA: Bovine serum albumin
CCK-8: Cell counting kit-8
CDNA: Complementary DNA
C-Fms: Colony stimulating factor-1 receptor
Ctsk: Cathepsin K
DAPI: Rhodamine-marked phalloidin and 4′6-diamidino-2-phenylindole
Dcstamp: Dendritic cell-specific transmembrane protein
DMSO: Dimethyl sulfoxide
ERK: Extracellular signal-regulated kinase
FBS: Fetal bovine serum
Fos: Proto-oncogene c-Fos
HE: Hematoxylin and eosin
IKK: IκB kinase
JNK: C-Jun N-terminal kinase
LM: LM22B-10
MAPK: Mitogen-activated protein kinase
M-CSF: Macrophage colony-stimulating factor
Micro-CT: Microscopic computed tomography
Mmp9: Matrix metalloproteinase
MPTPs: Mitochondria permeability transition pores
MRL: Myrislignan
MtROS: Mitochondrial ROS
NFATc1: Nuclear factor of activated T cells 1
NF-κB: Nuclear factor-κB
OD: Optical density
OS: Oxidative stress
OVX: Ovariectomy
PBS: Phosphate-buffered saline
PFA: Paraformaldehyde
PMSF: Phenylmethanesulfonyl fluoride
qRT-PCR: Quantitative reverse transcription-polymerase chain reaction
RANK: Receptor activator of nuclear factor κB
RANKL: Receptor activator of nuclear factor-κB ligand
ROS: Reactive oxygen species
SD: Standard deviation
SDS-PAGE: Sodium dodecyl sulfate–polyacrylamide gel electrophoresis
TBST: Tris buffered saline tween
TMRM: Tetramethyl rhodamine methyl ester
TRAF6: TNF receptor-associated factor 6
TRAP: Tartrate-resistant and phosphatase
α-MEM: Minimum essential medium alpha modification

## References

[1] Kim YS, Koh JM, Lee YS, Kim BJ, Lee SH, Lee KU (2009). Increased circulating heat shock protein 60 induced by menopause, stimulates apoptosis of osteoblast-lineage cells via up-regulation of toll-like receptors. Bone.

[2] Xiong J, Liao J, Liu X, Zhang Z, Adams J, Pacifici R (2022). A TrkB agonist prodrug prevents bone loss via inhibiting asparagine endopeptidase and increasing osteoprotegerin. Nat Commun.

[3] Wang F, Yang G, Li Y, Tang Z, Du J, Song H (2022). A peptide from wheat germ abolishes the senile osteoporosis by regulating OPG/RANKL/RANK/TRAF6 signaling pathway. Phytomedicine.

[4] Kobayashi K, Nojiri H, Saita Y, Morikawa D, Ozawa Y, Watanabe K (2015). Mitochondrial superoxide in osteocytes perturbs canalicular networks in the setting of age-related osteoporosis. Sci Rep.

[5] Yang Y, Feng N, Liang L, Jiang R, Pan Y, Geng N (2022). Progranulin, a moderator of estrogen/estrogen receptor alpha binding, regulates bone homeostasis through PERK/p-eIF2 signaling pathway. J Mol Med.

[6] Wu L, Liang J, Li J, Xu Y, Chen J, Su Y (2022). Onc201 reduces osteoclastogenesis and prevents ovariectomy-induced bone loss via inhibiting RANKL-induced NFATc1 activation and the integrin signaling pathway. Eur J Pharmacol.

[7] Qiu J, Jiang T, Yang G, Gong Y, Zhang W, Zheng X (2022). Neratinib exerts dual effects on cartilage degradation and osteoclast production in osteoarthritis by inhibiting the activation of the MAPK/NF-kappaB signaling pathways. Biochem Pharmacol.

[8] Wei L, Chen W, Huang L, Wang H, Su Y, Liang J (2022). Alpinetin ameliorates bone loss in LPS-induced inflammation osteolysis via ROS mediated P38/PI3K signaling pathway. Pharmacol Res.

[9] Salvadori L, Belladonna ML, Castiglioni B, Paiella M, Panfili E, Manenti T (2022). KYMASIN UP natural product inhibits osteoclastogenesis and improves osteoblast activity by modulating Src and p38 MAPK. Nutrients.

[10] Zhang Y, Wang J, Jing C, Zhou MX, Jin W, Yan X (2022). Purple tea water extract blocks RANKL-induced osteoclastogenesis through modulation of Akt/GSK3beta and Blimp1-Irf8 pathways. Food Funct.

[11] Ye C, Zhang W, Zhao Y, Zhang K, Hou W, Chen M (2022). Prussian blue nanozyme normalizes microenvironment to delay osteoporosis. Adv Healthc Mater.

[12] Laha D, Sarkar J, Maity J, Pramanik A, Howlader MSI, Barthels D (2022). Polyphenolic compounds inhibit osteoclast differentiation while reducing autophagy through limiting ROS and the mitochondrial membrane potential. Biomolecules.

[13] Zhang X, Jiang Y, Mao J, Ren X, Ji Y, Mao Y (2021). Hydroxytyrosol prevents periodontitis-induced bone loss by regulating mitochondrial function and mitogen-activated protein kinase signaling of bone cells. Free Radic Biol Med.

[14] Lee NK, Choi YG, Baik JY, Han SY, Jeong DW, Bae YS (2005). A crucial role for reactive oxygen species in RANKL-induced osteoclast differentiation. Blood.

[15] Hyeon S, Lee H, Yang Y, Jeong W (2013). Nrf2 deficiency induces oxidative stress and promotes RANKL-induced osteoclast differentiation. Free Radic Biol Med.

[16] Xu ZS, Wang XY, Xiao DM, Hu LF, Lu M, Wu ZY (2011). Hydrogen sulfide protects MC3T3-E1 osteoblastic cells against H2O2-induced oxidative damage-implications for the treatment of osteoporosis. Free Radical Bio Med.

[17] Lean JM, Davies JT, Fuller K, Jagger CJ, Kirstein B, Partington GA (2003). A crucial role for thiol antioxidants in estrogen-deficiency bone loss. J Clin Invest.

[18] Brand MD (2010). The sites and topology of mitochondrial superoxide production. Exp Gerontol.

[19] Callaway DA, Jiang JX (2015). Reactive oxygen species and oxidative stress in osteoclastogenesis, skeletal aging and bone diseases. J Bone Miner Metab.

[20] Goldberg M (2008). In vitro and in vivo studies on the toxicity of dental resin components: a review. Clin Oral Invest.

[21] Kim HJ, Lee DK, Jin X, Che X, Ryu SH, Choi JY (2022). Phospholipase D2 controls bone homeostasis by modulating M-CSF-dependent osteoclastic cell migration and microtubule stability. Exp Mol Med.

[22] Long F, Chen R, Su Y, Liang J, Xian Y, Yang F (2022). Epoxymicheliolide inhibits osteoclastogenesis and resists OVX-induced osteoporosis by suppressing ERK1/2 and NFATc1 signaling. Int Immunopharmacol.

[23] Kim HN, Ponte F, Nookaew I, Ucer Ozgurel S, Marques-Carvalho A, Iyer S (2020). Estrogens decrease osteoclast number by attenuating mitochondria oxidative phosphorylation and ATP production in early osteoclast precursors. Sci Rep.

[24] Suh KS, Chon S, Jung WW, Choi EM (2019). Crocin attenuates methylglyoxal-induced osteoclast dysfunction by regulating glyoxalase, oxidative stress, and mitochondrial function. Food Chem Toxicol.

[25] Li X, Wang Y, Li L, Zhou S, Zhao F (2021). Sclareol inhibits RANKL-induced osteoclastogenesis and promotes osteoblastogenesis through promoting CCN1 expression via repressing the MAPK pathway. Cell Biol Toxicol.

[26] Li X, Lin X, Wu Z, Su Y, Liang J, Chen R (2021). Pristimerin protects against OVX-mediated bone loss by attenuating osteoclast formation and activity via inhibition of RANKL-mediated activation of NF-kappaB and ERK signaling pathways. Drug Des Devel Ther.

[27] Javadov S, Jang S, Agostini B (2014). Crosstalk between mitogen-activated protein kinases and mitochondria in cardiac diseases: therapeutic perspectives. Pharmacol Ther.

[28] Chen K, Yan Z, Wang Y, Yang Y, Cai M, Huang C (2020). Shikonin mitigates ovariectomy-induced bone loss and RANKL-induced osteoclastogenesis via TRAF6-mediated signaling pathways. Biomed Pharmacother.

[29] Liu J, Ding S, Yang L, Zhao X, Ren R, Wang Y (2022). Integration of pharmacodynamics and metabolomics reveals the therapeutic effects of 6-acetylacteoside on ovariectomy-induced osteoporosis mice. Phytomedicine.

[30] Liu H, Gu R, Huang Q, Liu Y, Liu C, Liao S (2022). Isoliensinine suppresses osteoclast formation through NF-kappaB signaling pathways and relieves ovariectomy-induced bone loss. Front Pharmacol.

[31] Zhao Q, Zhang JL, Li F (2018). Application of metabolomics in the study of natural products. Nat Prod Bioprospect.

[32] Zhang J, Chen J, Lv K, Li B, Yan B, Gai L (2021). Myrislignan induces redox imbalance and activates autophagy in toxoplasma gondii. Front Cell Infect Microbiol.

[33] Zhang J, Si H, Li B, Zhou X, Zhang J (2019). Myrislignan exhibits activities against toxoplasma gondii RH strain by triggering mitochondrial dysfunction. Front Microbiol.

[34] Li J, Liang J, Wu L, Xu Y, Xiao C, Yang X (2022). CYT387, a JAK-specific inhibitor impedes osteoclast activity and oophorectomy-induced osteoporosis via modulating RANKL and ROS signaling pathways. Front Pharmacol.

[35] Chen F, Tian L, Pu X, Zeng Q, Xiao Y, Chen X (2022). Enhanced ectopic bone formation by strontium-substituted calcium phosphate ceramics through regulation of osteoclastogenesis and osteoblastogenesis. Biomater Sci.

[36] He J, Zheng L, Li X, Huang F, Hu S, Chen L (2023). Obacunone targets macrophage migration inhibitory factor (MIF) to impede osteoclastogenesis and alleviate ovariectomy-induced bone loss. J Adv Res.

[37] Liu Y, Wang C, Wang G, Sun Y, Deng Z, Chen L (2019). Loureirin B suppresses RANKL-induced osteoclastogenesis and ovariectomized osteoporosis via attenuating NFATc1 and ROS activities. Theranostics.

[38] Huong LT, Gal M, Kim O, Tran PT, Nhiem NX, Kiem PV (2022). 23-Hydroxyursolic acid from viburnum lutescens inhibits osteoclast differentiation in vitro and lipopolysaccharide-induced bone loss in vivo by suppressing c-Fos and NF-kappaB signalling. Int Immunopharmacol.

[39] Kim HJ, Kim BK, Ohk B, Yoon HJ, Kang WY, Cho S (2019). Estrogen-related receptor gamma negatively regulates osteoclastogenesis and protects against inflammatory bone loss. J Cell Physiol.

[40] Sun W, Guo S, Li Y, Li J, Liu C, Chen Y (2022). Anoctamin 1 controls bone resorption by coupling Cl(-) channel activation with RANKL-RANK signaling transduction. Nat Commun.

[41] Jin F, Zhu Y, Liu M, Wang R, Cui Y, Wu Y (2022). Babam2 negatively regulates osteoclastogenesis by interacting with Hey1 to inhibit Nfatc1 transcription. Int J Biol Sci.

[42] Liu S, Du J, Li D, Yang P, Kou Y, Li C (2020). Oxidative stress induced pyroptosis leads to osteogenic dysfunction of MG63 cells. J Mol Histol.

[43] Bhattarai G, Poudel SB, Kook SH, Lee JC (2017). Anti-inflammatory, anti-osteoclastic, and antioxidant activities of genistein protect against alveolar bone loss and periodontal tissue degradation in a mouse model of periodontitis. J Biomed Mater Res A.

[44] Chen YH, Peng SY, Cheng MT, Hsu YP, Huang ZX, Cheng WT (2019). Different susceptibilities of osteoclasts and osteoblasts to glucocorticoid-induced oxidative stress and mitochondrial alterations. Chin J Physiol.

[45] Jimi E, Katagiri T (2022). Critical roles of NF-kappaB signaling molecules in bone metabolism revealed by genetic mutations in osteopetrosis. Int J Mol Sci.

[46] Fu X, Sun X, Zhang C, Lv N, Guo H, Xing C (2022). Genkwanin prevents lipopolysaccharide-induced inflammatory bone destruction and ovariectomy-induced bone loss. Front Nutr.

[47] Zhang Y, Wang H, Zhu G, Qian A, Chen W (2020). F2r negatively regulates osteoclastogenesis through inhibiting the Akt and NFkappaB signaling pathways. Int J Biol Sci.

[48] Xian Y, Su Y, Liang J, Long F, Feng X, Xiao Y (2021). Oroxylin A reduces osteoclast formation and bone resorption via suppressing RANKL-induced ROS and NFATc1 activation. Biochem Pharmacol.

[49] Liu T, Jiang L, Xiang Z, Li J, Zhang Y, Xiang T (2022). Tereticornate A suppresses RANKL-induced osteoclastogenesis via the downregulation of c-Src and TRAF6 and the inhibition of RANK signaling pathways. Biomed Pharmacother.

[50] Vacek TP, Kalani A, Voor MJ, Tyagi SC, Tyagi N (2013). The role of homocysteine in bone remodeling. Clin Chem Lab Med.

[51] Yuan L, Zhao N, Wang J, Liu Y, Meng L, Guo S (2021). Major vault protein (MVP) negatively regulates osteoclastogenesis via calcineurin-NFATc1 pathway inhibition. Theranostics.

[52] Srinivasan S, Avadhani NG (2007). Hypoxia-mediated mitochondrial stress in RAW264.7 cells induces osteoclast-like TRAP-positive cells. Ann N Y Acad Sci.

[53] Jie Z, Shen S, Zhao X, Xu W, Zhang X, Huang B (2019). Activating beta-catenin/Pax6 axis negatively regulates osteoclastogenesis by selectively inhibiting phosphorylation of p38/MAPK. FASEB J.

[54] Tang L, Wu M, Lu S, Zhang H, Shen Y, Shen C (2021). Fgf9 negatively regulates bone mass by inhibiting osteogenesis and promoting osteoclastogenesis via MAPK and PI3K/AKT signaling. J Bone Miner Res.

[55] Huang L, Chen W, Wei L, Su Y, Liang J, Lian H (2022). Lonafarnib inhibits farnesyltransferase via suppressing ERK signaling pathway to prevent osteoclastogenesis in titanium particle-induced osteolysis. Front Pharmacol.

[56] Yu J, Kim S, Lee N, Jeon H, Lee J, Takami M (2021). Pax5 negatively regulates osteoclastogenesis through downregulation of blimp1. Int J Mol Sci.

[57] Chen X, Li C, Cao X, Jia X, Chen X, Wang Z (2022). Mitochondria-targeted supramolecular coordination container encapsulated with exogenous itaconate for synergistic therapy of joint inflammation. Theranostics.

[58] Gal M, Kim O, Tran PT, Huong LT, Nhiem NX, Van Kiem P (2022). Mussaendoside O, a N-triterpene cycloartane saponin, attenuates RANKL-induced osteoclastogenesis and inhibits lipopolysaccharide-induced bone loss. Phytomedicine.

[59] Cheon YH, Lee CH, Kim S, Park GD, Kwak SC, Cho HJ (2021). Pitavastatin prevents ovariectomy-induced osteoporosis by regulating osteoclastic resorption and osteoblastic formation. Biomed Pharmacother.

[60] Chen K, Qiu P, Yuan Y, Zheng L, He J, Wang C (2019). Pseurotin A inhibits osteoclastogenesis and prevents ovariectomized-induced bone loss by suppressing reactive oxygen species. Theranostics.

[61] Tao H, Tao Y, Yang C, Li W, Zhang W, Li X (2022). Gut metabolite urolithin a inhibits osteoclastogenesis and senile osteoporosis by enhancing the autophagy capacity of bone marrow macrophages. Front Pharmacol.

[62] Cho E, Cheon S, Ding M, Lim K, Park SW, Park C (2022). Identification of novel genes for cell fusion during osteoclast formation. Int J Mol Sci.

[63] Mukhopadhyay S, Vander Heiden MG, McCormick F (2021). The metabolic landscape of RAS-driven cancers from biology to therapy. Nat Cancer.

