# Supplementary material for: Myrislignan targets extracellular signal-regulated kinase (ERK) and modulates mitochondrial function to dampen osteoclastogenesis and ovariectomy-induced osteoporosis
Source: J Transl Med. 2023 Nov 22;21:839. doi: 10.1186/s12967-023-04706-2 (PMC10664306; doi:10.1186/s12967-023-04706-2)
Supplement: Supplementary file 1 — Additional file 1: Figure S1. MRL inhibits RANKL-induced NFATc1 expression in vitro. (a) After being stimulated with 50 ng/ml RANKL for five days and 30 μM MRL for 0, 1, 3, and 5 days, the expression of osteoclast-related downstream signaling proteins was detected by Western Blotting. (b-e) Quantitative data of band intensity ratios of c-FOS, NFATc1, CTSK, and Atp6v0d2 relative to β-actin. Figure S2. LM has a negative toxic effect on BMMs. (a) BMMs cell proliferation was detected by CCK-8 assay after treatment with different concentrations of LM for 48 hours. (b) Cell proliferation of BMMs was detected by CCK-8 assay after being unitedly treated with 30 μM MRL and different concentrations of LM. (c) Representative images of TRAP staining showed that after stimulation with 50 ng/ml RANKL for seven days, osteoclast differentiation was activated by 30 μM LM but was suppressed by 30 μM MRL. (d) Quantitative data of TRAP-positive osteoclasts per well was shown. Figure S3. The weight of OVX-induced mice. (a) Verifying the successful construction of the OVX-induced model was done by measuring the body weight of the mice. [file 12967_2023_4706_MOESM1_ESM.docx]

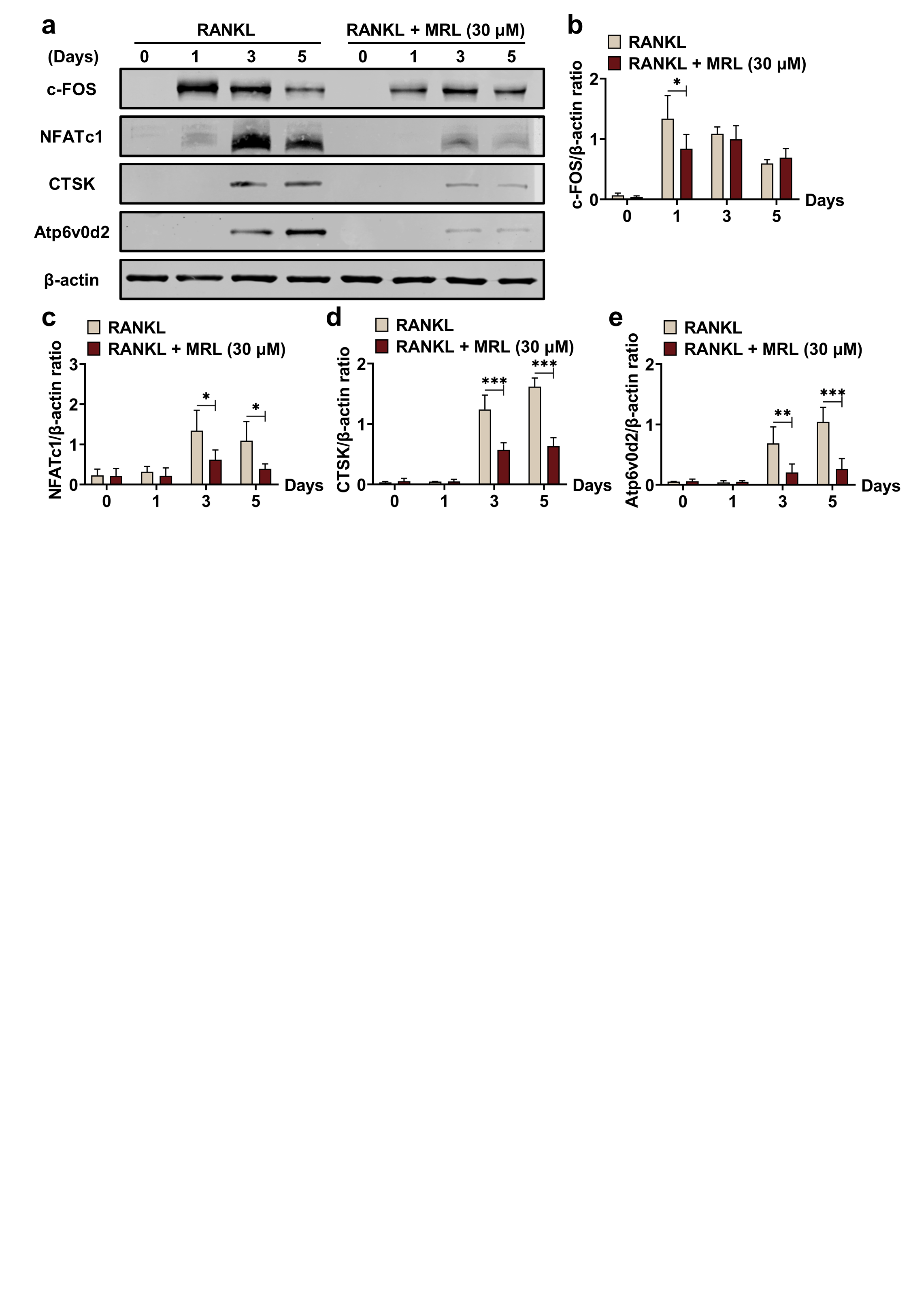
**Myrislignan Targets Extracellular Signal-regulated Kinase (ERK) and Modulates Mitochondrial Function to Dampen Osteoclastogenesis and Ovariectomy-induced Osteoporosis**

**Additional file 1: Fig. S1 MRL inhibits RANKL-induced NFATc1 expression *in vitro*. a** After being stimulated with 50 ng/ml RANKL for five days and 30 μM MRL for 0, 1, 3, and 5 days, the expression of osteoclast-related downstream signaling proteins was detected by Western Blotting. **b-e** Quantitative data of band intensity ratios of c-FOS, NFATc1, CTSK, and Atp6v0d2 relative to β-actin (n=3 for each group). All the data were expressed as mean ± SD. **p* < 0.05, ***p* < 0.01 and ****p* < 0.001.


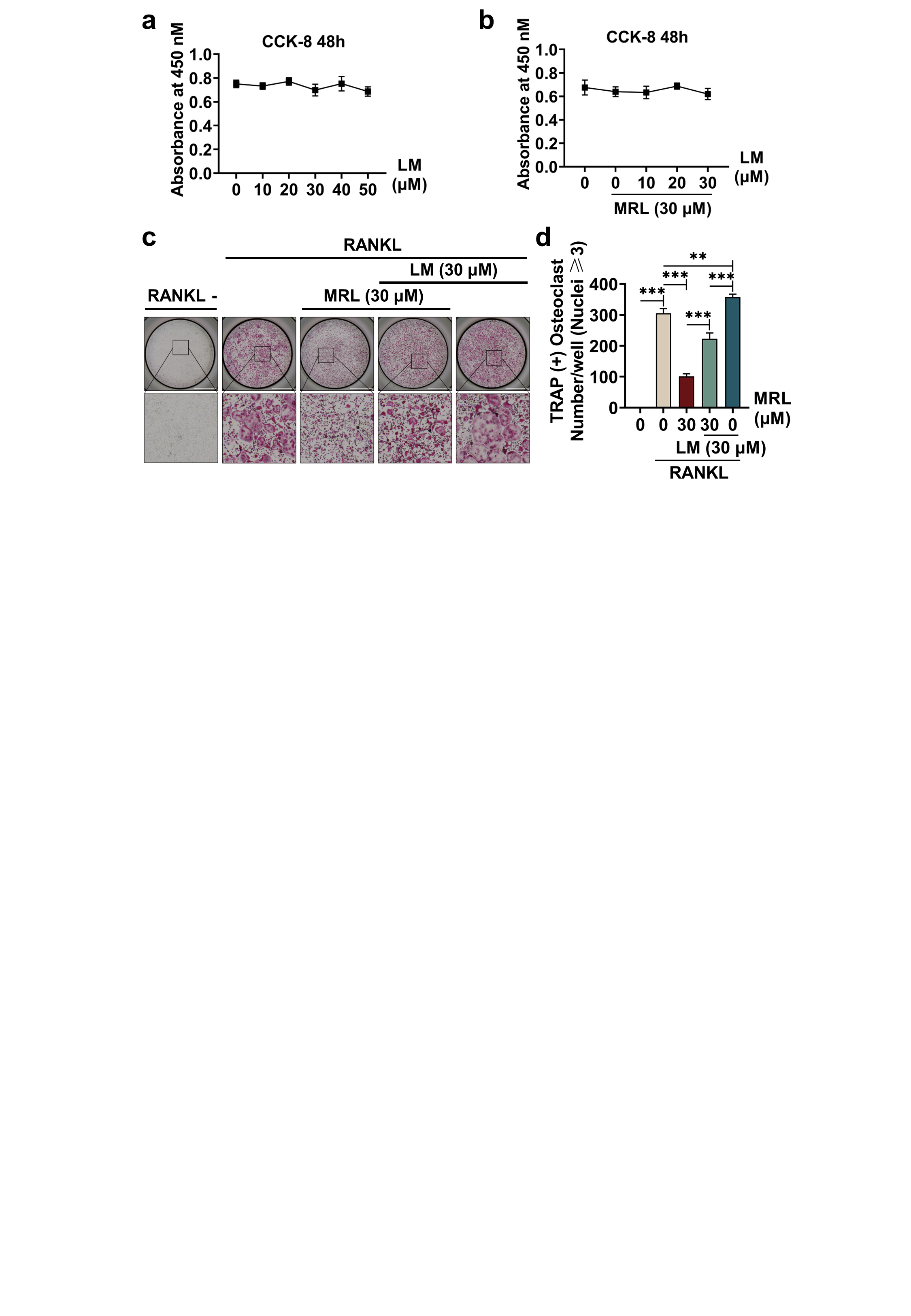
**Additional file 1: Fig. S2 LM has a negative toxic effect on BMMs. a** BMMs cell proliferation was detected by CCK-8 assay after treatment with different concentrations of LM for 48 hours (n=3 for each group). **b** Cell proliferation of BMMs was detected by CCK-8 assay after being unitedly treated with 30 μM MRL and different concentrations of LM (n=3 for each group). **c** Representative images of TRAP staining showed that after stimulation with 50 ng/ml RANKL for seven days, osteoclast differentiation was activated by 30 μM LM but was suppressed by 30 μM MRL. Scale bar = 2,000 μm. **d** Quantitative data of TRAP-positive osteoclasts per well was shown (n=3 for each group). All the data were expressed as mean ± SD. **p* < 0.05, ***p* < 0.01 and ****p* < 0.001.


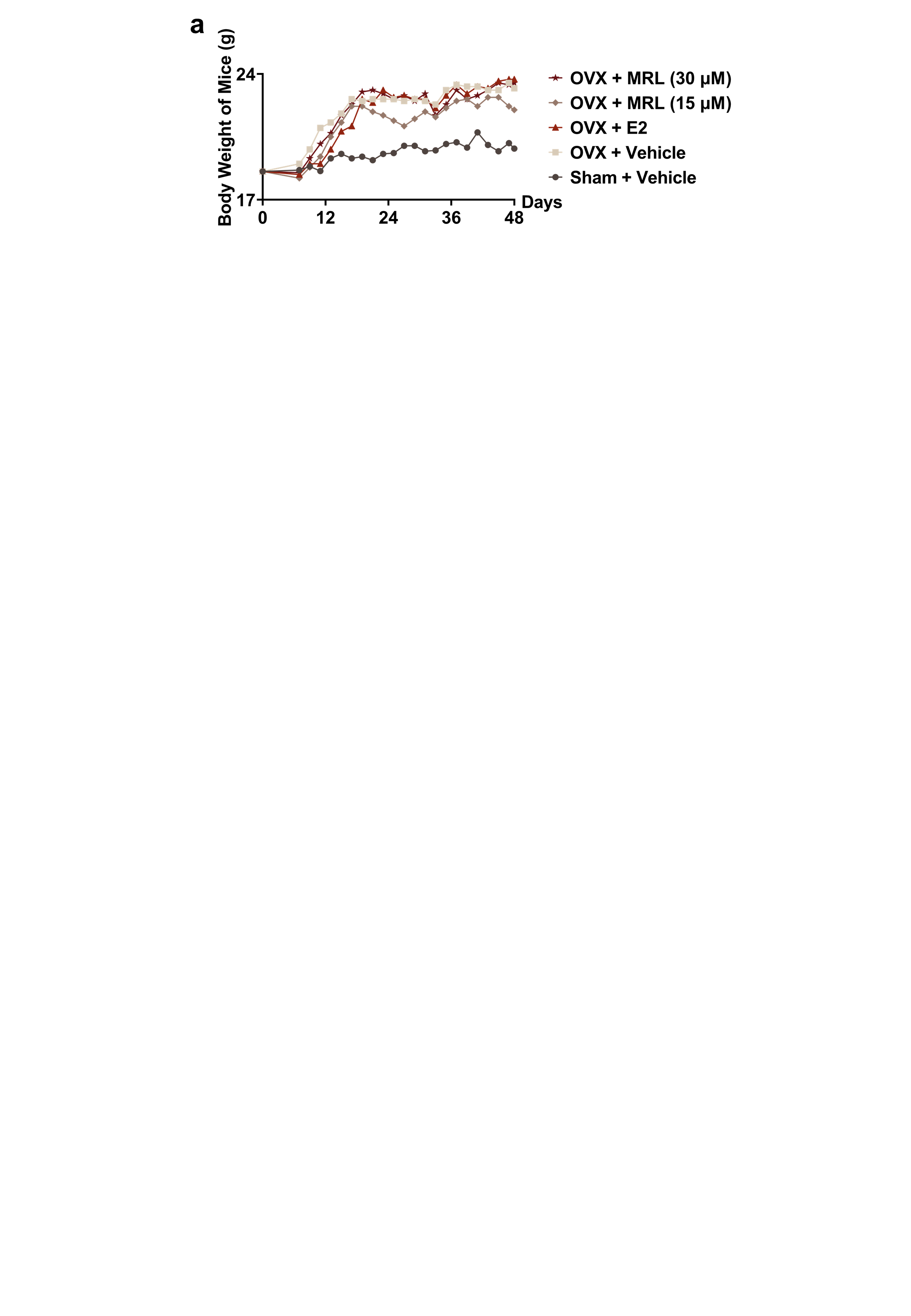
**Additional file 1: Fig. S3 The weight of OVX-induced mice. a** Verifying the successful construction of the OVX-induced model was done by measuring the body weight of the mice.
